# Feasibility and Comparative Effectiveness for the Delivery of the National Diabetes Prevention Program through Cooperative Extension in Rural Communities

**DOI:** 10.3390/ijerph19169902

**Published:** 2022-08-11

**Authors:** Anna M. Gorczyca, Richard A. Washburn, Patricia Smith, Robert N. Montgomery, Lyndsie M. Koon, Mary Hastert, Kameron B. Suire, Joseph E. Donnelly

**Affiliations:** 1 Division of Physical Activity and Weight Management, Department of Internal Medicine, University of Kansas Medical Center, Kansas City, KS 66160, USA; 2 Department of Biostatistics and Data Science, University of Kansas Medical Center, Kansas City, KS 66160, USA; 3 Research and Training Center on Independent Living, University of Kansas, Lawrence, KS 66045, USA

**Keywords:** national diabetes prevention program, rural, cooperative extension, physical activity, weight loss

## Abstract

The U.S. Cooperative Extension Service (CE) has potential to deliver the National Diabetes Prevention Program (NDPP) to rural residents with prediabetes. However, the CE remains underutilized for the delivery of NDPP. We compared the feasibility/effectiveness of the NDPP (0–6 mos.) delivered by CE personnel to rural residents with prediabetes using Zoom^®^ (CE-Zoom^®^) or by our research staff using Facebook^®^ (FB). Adults (n = 31, age ~55 years) were enrolled (CE-Zoom^®^ n = 16, FB n = 15). Attendance did not differ significantly between groups (CE Zoom^®^ = 69%, FB = 83%, *p* = 0.15). Participant retention was similar in the CE Zoom^®^ (88%) and FB groups (87%). CE-Zoom^®^ and FB^®^ groups provided weekly, self-monitoring data for 83% and 84% of the 24 potential weeks, respectively. Six-month weight loss was not different between groups (CE-Zoom^®^ = −5.99 ± 8.0 kg, −5.4%, FB = −1.68 ± 3.3 kg, −1.6% *p* = 0.13). Participants achieving ≥5% weight loss was greater in the CE-Zoom^®^ (44%) compared with the FB group (7%, *p* = 0.04). Participants achieving the NDPP program goal for physical activity (≥150 min/week) did not differ (CE-Zoom^®^ = 75%, FB = 67%, *p* = 0.91). This pilot trial demonstrated the potential feasibility and effectiveness of the NDPP delivered by CE personnel in a group remote format (Zoom^®^) to adults with prediabetes living in rural areas.

## 1. Introduction

The National Diabetes Prevention Program (NDPP), administered by the Centers for Disease Control (CDC), provides the framework for the prevention of type 2 diabetes (T2D) in the U.S. [1]. The effectiveness of the NDPP, which represents a lower-cost, scalable translation of the intensive lifestyle intervention utilized in the original Diabetes Prevention trial [2], has been demonstrated [3,4]. Currently, the NDPP is delivered in-person, online, distance learning or via combined formats to groups or individual participants by lifestyle coaches from a variety of educational backgrounds following completion of CDC-approved training. The NDPP lifestyle intervention includes a minimum of 16 core sessions over 6 months and 6 monthly maintenance sessions. The curriculum recommends a low fat/reduced calorie diet and increased moderate-to-vigorous intensity physical activity (MVPA) to meet 12-month goals of achieving ≥5% weight loss and ≥150 min/week. MVPA. Sessions are designed to improve self-efficacy and problem-solving skills as week as to provide social support and strategies to assist with meeting the 12-month goals [1]. Although CDC recognized NDPP programs, which satisfy requirements for interventionist training, fidelity, and effectiveness, are available in all 50 states and ~28% of counties in the U.S., it is estimated that all organizations offering the NDPP had reached only 297,065 eligible adults through January 2019, or less that 0.4% of U.S. adults with prediabetes. This suggests the need for strategies to expand the reach of the NDPP. 

**Figure 1:**
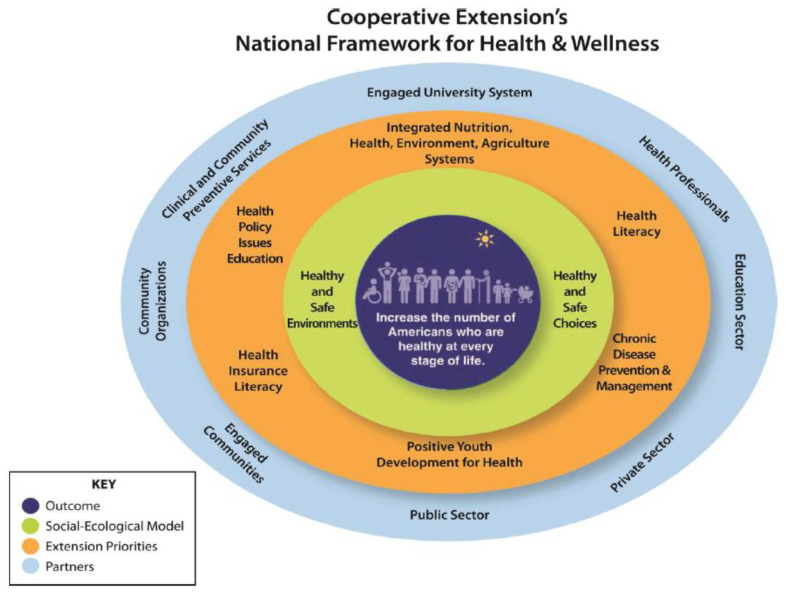
Cooperative Extension’s National Framework for Health and Wellness. Note: This framework uses an adapted social-ecological model to present the interplay between outcome, goal, environments, priorities and partnerships. Reprinted with permission from Ref. [7]. Copyright 2015 Extension Journal, Inc.

The 60 million residents of rural areas represent an underserved population with a prevalence of T2D that is ~17% higher than in urban areas, and thus could benefit from improved access to the NDPP. However, the availability of DPP sites is significantly lower in rural (14.6%) compared with urban counties (48.4%) [5]. The U.S. Cooperative Extension (CE), a partnership between the U.S Department of Agriculture, land-grant universities, and state and county government, maintains ~3200 offices across the U.S. The CE offices have the physical space and employ nutrition and consumer science professionals capable of delivering research-based interventions such as the NDPP. Such efforts would be consistent with the CE priority for chronic disease prevention and management as outlined in the 2014 Cooperative Extension’s National Framework for Health and Wellness (Figure 1) [6,7]. However, the CE is underutilized for the delivery of NDPP to adults with prediabetes living in rural areas. Currently the CDC website list only 20 NDPP programs across 16 states that are delivered through the CE (13 full recognition, 5 preliminary recognition, and 2 unrecognized). Additionally, in 2021 the Cooperative Extension’s National Framework for Health Equity and Well-Being was released with specific recommendations to increase health equity as a core system value, improve community assessments, and establish partnerships with academic units, universities, and the public and private sectors to reduce health inequalities, including people of color and those living in rural communities [8]. 

The feasibility and comparative effectiveness of different strategies for delivering the NDPP to rural residents have not been evaluated. Therefore, this pilot trial compared the feasibility and effectiveness of the intensive intervention component (0–6 months) of the NDPP lifestyle intervention. One was delivered by CE personnel to groups of rural residents with prediabetes using Zoom^®^ (Zoom Video Communications, Inc., San Jose, CA, USA) and another with the same intervention was delivered by a research staff member via a private Facebook^®^ (Meta Platforms, Inc., Menlo Park, CA, USA) group.

## 2. Materials and Methods

Overview: NDPP Lifestyle intervention. This trial delivered the 6 months (24 sessions) weight loss portion of the 12-month NDPP Prevent T2 curriculum. This intervention is based on Social Cognitive Theory and includes topics on nutrition and physical activity in addition to behavior change strategies, including self-monitoring, environmental control, positive reinforcement, and accountability [9]. The curriculum stresses goal setting related to modest weight loss (5–7%) and increases in fruit and vegetable consumption and physical activity. The physical activity portion of the curriculum emphasizes incorporating both light and MVPA, e.g., taking the stairs, stretching, gardening, and parking further away at the grocery store, etc. to achieve the 150 min/week goal for MVPA. In this trial, participants were asked to self-monitor body weight using a (Fitbit Aria Air^TM^ scale) (Google LLC, Mountain View, CA, USA) minutes of MVPA (Fitbit Versa 2) and fruit and vegetable intake on paper charts and to enter all self-monitoring data on a REDCap form that was emailed to participants weekly. Weekly de-identified, self-monitoring data were collated and presented to participants in both the Zoom^®^ and Facebook^®^ groups. The data were used by lifestyle coaches to provide feedback and promote discussion regarding the successes and challenges over the previous week. 

Intervention sites. Research staff worked with administrators from Kansas State Research and Extension to identify two extension offices located in rural Kansas counties with interest in participating in this project. Both CE offices agreed to work with our research staff on participant recruitment while the CE office that was designated to deliver the group-based Zoom^®^ intervention agreed to identify a member of their staff to serve as the lifestyle coach. 

Participant recruitment/compensation. Research staff worked with both participating CE offices to recruit participants through list serves maintained by the CE offices, social, traditional media (newspaper-newsletters), local businesses, and physicians’ offices. Participants were informed of their group assignment at their baseline testing appointment. This trial was approved by the University’s Institutional Review Board and registered on clinical trials.gov (NCT05387434). All participants provided informed consent prior to study initiation. Participants were compensated $20 for time and travel to attend each of the three outcome testing visits (baseline, 3, and 6 months) and allowed to keep the Fitbit and wireless scales used for self-monitoring. This project was completed between March 2021 and April 2022. 

Inclusion/Exclusion criteria. Inclusion: (1) Age ≥ 18 years. (2) Prediabetes defined as self-reported HbA1c = 5.7–6.4% or fasting glucose = 100–125 mg/dL or history of gestational diabetes mellitus or a score of ≥5 on the CDC-NDPP prediabetes screener. (3) Ability to commit to weekly participation in either a Zoom^®^ or Facebook^®^ intervention over 6 months. (4) Live in the rural county served by the participating CE office. (5) Have access to the internet and have the capability/capacity to use Zoom^®^ and Facebook^®^. (6) Clearance from primary care physician. Exclusion. (1) Diagnosis of T2D. (2) The inability to participate in MVPA. 

Lifestyle Coach Training. A CE family and consumer science agent served as the lifestyle coach for the Zoom^®^ delivered group while a member of our research staff served as the lifestyle coach for the Facebook^®^ group. Both lifestyle coaches completed the CDC-NDPP training conducted by Telligen^®^ Inc. (West Des Moines, IA, USA) and completed follow-up training conducted by the research team. To ensure interventionist availability in the event of illness, job change etc., both interventionists completed training on the Zoom^®^ and Facebook^®^ interventions.

Telligen^®^ is one of twelve entities with a signed memorandum of understanding with the CDC to provide NDPP lifestyle coach training. The training consisted of 6-h. sessions over 2 days conducted remotely via WebEx^®^ (San Jose, CA, USA). Telligen^®^ NDPP training includes problem-based learning, the transtheoretical model, motivational interviewing, and techniques for group facilitation. Successful completion of a practical exam, which includes delivery of a sample lesson and group facilitation completed at the end of day 2 of training, was required for both lifestyle coaches. 

Following successful completion of the Telligen^®^ training, the research team provided a half-day virtual training session (Zoom^®^) for both lifestyle coaches to review the NDPP Prevent T2 curriculum and address any questions precipitated by completion of the Telligen^®^ training. They also completed additional four, two-hour Zoom^®^ sessions to review procedures specific to each intervention group. Lifestyle coaches were asked to listen to audio recordings from a previously completed, remotely delivered, group-based, NIH-funded weight management trial (DK108732) These recordings demonstrated effective leadership and facilitation techniques for conducting remote group-based intervention sessions. Prior to initiation of the intervention, lifestyle coaches were required to successfully conduct a minimum of two simulated sessions with members of the research staff serving as participants. All training and simulated sessions were observed by the study coordinator who used a standardized fidelity checklist to evaluate training skills. 

CE-Zoom^®^ group. Participants were asked to attend weekly 60-min group sessions from a private area in their homes or other private location where internet access was available. Participants were allowed to join the Zoom^®^ meeting using video or voice only. Zoom^®^ sessions were conducted in the early evening on a weekday (M-Th) depending on the lifestyle coach and participant availability. Participants received an email with a Zoom^®^ meeting link and a reminder to submit their self-monitoring data via the REDCap form on the morning of each scheduled meeting. Each session included a check-in question to stimulate group discussion, e.g., what was your biggest challenge in meeting your physical activity goal, review of self-monitoring data (weight, physical activity, and diet), review of the homework assignment from the previous session, presentation and discussion of a weekly topic, and a session summary. Participants missing two consecutive sessions were contacted by the lifestyle coach (email/phone/text) to remind them of the importance of attendance for program success. Participants were provided with a notebook that included handouts/worksheets/assignments specific to each of the 24 weekly lessons. They were provided with weekly charts to self-monitor weight, physical activity, and fruit and vegetable consumption. 

Facebook^®^ group. Participants were asked to join a private, lifestyle-coach moderated, Facebook^®^ group where the content was accessible only to invited group members; the existence of the page was hidden from the public. A set of ground rules and a code of conduct for utilizing the Facebook^®^ group page were distributed to participants. Participants were required to agree to disclaimers and limits of liability associated with the use of Facebook^®^ and were advised to adjust privacy settings to control access by other members of the private Facebook^®^ group. 

Every Monday morning across the 6-month intervention, the lifestyle coach posted a new module from the Prevent T2 curriculum to Facebook^®^. On Tuesday mornings, the lifestyle coach shared de-identified participant self-monitoring data (diet, physical activity, and weight change) and a brief comment on group performance to allow participants to assess their progress relative to others in the group. On Wednesday and Friday mornings, the lifestyle coach posted brief pre-scripted discussion questions such as, “What strategies to increase your level of PA will you try this week?” that were designed to reinforce the primary objectives of each module and to facilitate inter-participant discussion around these topics.

Participants were asked to comment on each of the discussion point posts. The lifestyle coach monitored the responses to the discussion question posts daily and answered questions and/or corrected any misinformation in the participants’ posts. The Friday morning post included a reminder to sync self-monitoring devices by midnight on Sunday. New posts were “pinned” to the top of the Facebook^®^ wall and included in the “announcements section” to ensure they were easily identified. The lifestyle coach contacted participants not active/engaged with two consecutive modules by Facebook^®^ by private message to remind them of the importance of active participation for program success. Facebook^®^ interactions were tracked across the trial in the form of “liking” or “commenting” on the posts or by contributing new posts [10]. All tracking was completed by a member of the research staff by conducting a manual review of the previous week’s Facebook^®^ group activity. 

Quality control. All Zoom^®^ sessions were audio recorded and saved to a secure server. The study coordinator randomly reviewed one Zoom^®^ session per month using a standardized checklist of topics to be covered. Based on this checklist, the lifestyle coach delivered over 80% of scheduled content, which met our predetermined standard for intervention quality. The study coordinator also monitored the delivery of the Facebook^®^ intervention to ensure that weekly Prevent T2 modules and discussion questions were posted on schedule. 

### 2.1. Outcome Assessments

Feasibility. Recruitment. Participant recruitment source was assessed on the initial participant eligibility screener. Qualitative data regarding recruitment were obtained from a focus group with 13 participants that was conducted following the completion of the 6-month intervention. Attendance for the CE Zoom^®^ group was defined as being present at the beginning and end of the Zoom meeting as assessed by lifestyle coach records. Attendance for the Facebook^®^ group was defined as having accessed the weekly Prevent T2D module, i.e., the participant viewed the module within one week of posting for the Facebook^®^ group. Participant retention was defined as completing outcome testing at 3 and 6 months. The feasibility of self-monitoring was assessed as the percentage of weeks participants submitted self-monitoring data for weight and MVPA averaged across the 6-month intervention. Safety was assessed as the percentage of participants reporting an adverse event considered to be associated with participation in the intervention. 

Effectiveness. Changes in body weight (kg and percent) and body mass index (BMI) across 6 months were the primary outcomes used to evaluate intervention effectiveness. All assessments, with the exception of MVPA, were conducted at the participating CE offices by members of our research staff. Weight and height were assessed between 6 and 11 a.m. with participants in a standard hospital gown following a minimum of a 12 h fast. Body weight was assessed to the nearest 0.1 kg with a calibrated digital scale (Belfour Inc., Model #PS6600, Saukville, WI, USA). Standing height was assessed to the nearest cm using a stadiometer (Model PE-WM-60–84, Perspective Enterprises, Portage, MI, USA) and used in the calculation of BMI (kg/m^2^). We also assessed the percentage of participants achieving the ≥150 min/week goal for MVPA as a measure of effectiveness. 

Participant satisfaction. Following completion of the interventions, participants in both intervention groups were asked to complete a REDCap survey regarding several aspects of the interventions, including overall program satisfaction, satisfaction with the lifestyle coach, program length, and problems with internet connectivity, etc. 

### 2.2. Statistical Analysis 

Baseline measures and demographic characteristics were summarized for the complete sample and by group using means and standard deviations for continuous variables and frequencies and percentages for categorical variables. Group changes for weight (kg, percent) and BMI across 6 months were evaluated using ANCOVA. Fisher’s exact test and a test of proportions were used to compare the proportion of participants achieving clinically relevant weight loss (≥5%) at 6 months, and the proportion of participants achieving ≥150 min/week of MVPA and providing self-monitoring data averaged across 6 months, respectively. Pearson correlations were used to examine the association between session attendance and weight change for the CE-Zoom^®^ group and engagement with the Facebook^®^ intervention and weight loss in the Facebook^®^ group. Statistical analyses were conducted using SAS 9.4 (SAS Institute, Cary, NC, USA) and R 4.2.1 (RStudio, Boston, MA, USA). 

## 3. Results

**Figure 2:**
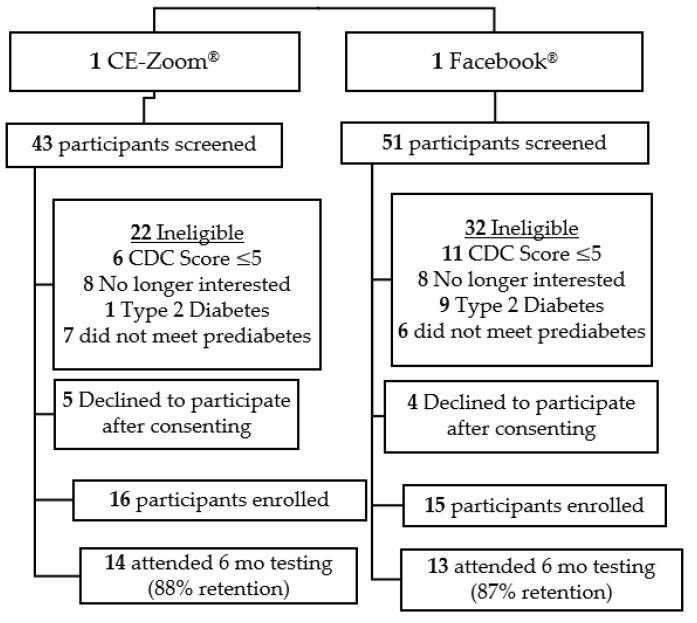
Consort Diagram.

Participants. A total of 94 adults with prediabetes were screened for eligibility (Figure 2). Thirty-one participants were enrolled in the intervention (CE Zoom^®^ = 16, Facebook^®^ = 15). Primary reasons for exclusion were CDC-NDPP prediabetes screener score ≤ 5 (31%), lab values (fasting glucose, oral glucose tolerance test, or HbA1c) that did not meet pre-diabetes criteria (30%), diagnosis of Type 2 diabetes (19%), or loss of interest in participation following completion of the screening process (23%). At baseline, participants were ~55 years old, 81% female, and 16% minority with an average BMI of ~36 kg/m^2^ (Table 1). 

Feasibility. Participant recruitment was completed in three months. Most participants (~33%) were recruited directly through engagement from the extension office, e.g., emails, newsletters, Facebook^®^ posts, or by word of mouth (~20%). Other recruitment strategies included emails and flyers directed to local healthcare providers and employers and advertisements in local newspapers and Facebook^®^ (Table 2). Attendance across 6 months was 76% and did not differ significantly between the CE Zoom^®^ (69%) and Facebook^®^ groups (83%, *p* = 0.15). Participant retention at 6 months was high and similar in the CE Zoom^®^ (n = 14, 88%) and Facebook^®^ groups (n = 12, 87%). Participants in the CE-Zoom ^®^ and Facebook^®^ groups provided weekly self-monitoring data across the 6-month intervention for 83% and 84%, of the 24 potential weeks, respectively. No adverse events were reported across the 6-month intervention. 

Effectiveness. Mean weight loss across 6 months did not differ significantly between the CE- Zoom^®^ (−5.99 ± 8.0 kg, −5.4%) and Facebook^®^ groups (−1.68 ± 3.3 kg, −1.6%) (mean diff = −3.7 kg, *p* = 0.13) (Table 3). The proportion of participants achieving ≥ 5% weight loss was significantly greater in the CE-Zoom^®^ (44%) compared with the Facebook^®^ group (7%, *p* = 0.04). Similar to the result for change in body weight, no significant group differences across 6 months were observed for change in BMI (CE-Zoom^®^ = −2.21 ± 2.87 kg/m^2^, Facebook^®^ = −0.70 ± 1.09 kg/m^2^) (mean diff = −1.29 kg/m^2^, *p* = 0.14). In the CE-Zoom^®^ group, higher attendance at weekly group sessions was associated with greater weight loss across 6 months (r = −0.56, *p* = 0.04). However, in the Facebook^®^ group, no associations between participant engagement defined as viewing posted Prevent T2 modules or interactions, i.e., “liking” (r = −0.40, *p* = 0.18) or “commenting” (r = −0.49, *p* = 0.08) on the posts or contributing to new posts (r = −0.56, *p* = 0.04); weight loss across 6 months were observed. Mean weekly self-reported MVPA across 6 months was similar in the CE-Zoom^®^ (196 ± 93 min/week) and Facebook^®^ groups (253 ± 143 min/week, *p* = 0.20). The percentage of participants achieving the NDPP program goal for MVPA (≥150 min/week) was high and did not differ significantly by intervention group (CE-Zoom^®^ = 75%, Facebook^®^ = 67%, *p* = 0.91). 

Participant satisfaction. Overall satisfaction with the intervention was similar in the CE-Zoom^®^ (Very satisfied/satisfied = 80%) and Facebook^®^ groups (Very satisfied/satisfied = 85%) (Table 4). Satisfaction with the lifestyle coach was also similar in the CE-Zoom^®^ (Very satisfied/satisfied = 80%) and Facebook^®^ groups (Very satisfied/satisfied = 87%). Eighty percent of participants reported increasing their physical activity and 87% reported making changes to their diet in the CE-Zoom^®^ group, which was similar to the 85% of participants who reported increasing their physical activity and 100% reported making changes to their diet in the Facebook^®^ group. Eighty-seven percent of participants in the CE-Zoom^®^ group and ninety-two percent of participants in the Facebook^®^ group indicated they would recommend the NDPP to a friend or family member. 

## 4. Discussion 

The ability to recruit and retain participants, the levels of intervention attendance, participant compliance with recommendations for self-monitoring, the reductions in body weight, and increases in physical activity observed in this pilot trial suggest the feasibility and potential effectiveness of utilizing the CE for delivery of the NDPP to adults with prediabetes living in rural areas. 

Participant recruitment strategies successfully employed to recruit rural adults in the current trial, including invitation letters/mailers/flyers, emails, newspaper, and Facebook^®^ advertising are similar to those that have been successful in recruiting NDPP participants from primarily urban/suburban areas [11] and are typical of traditional or passive recruitment methods. These strategies were also successful in recruiting a small sample of rural participants through the CE over a period of 2–3 months. However, the success of these passive strategies in recruiting larger numbers of rural participants and the potential of including collaborations with rural healthcare providers and other community partners on improving participant recruitment and retention through CE are unknown and worthy of investigation. A recent trial testing the effectiveness of a commercial digital-DPP [12] used the population health management approach, which entailed an electronic health record query with local healthcare providers, an initial physician review of patients identified, sending a letter invitation with an option to mail back an ‘opt-out of care’ postcard, and then a telephone follow-up for those not opting out [13]. This method of recruitment is considered active [14,15] and may have the potential to increase the diversity and representativeness of the target population the NDPP is trying to reach. 

Weight loss across 6 months in the CE-Zoom^®^ group in this trial (−5.9 kg, −5.4%, 44% ≥5%) is similar or slightly better than other published data describing the effectiveness of the NDPP in primarily urban/suburban populations [16,17]. For example, Eaglehouse et al. [17] reported −5.7% weight loss at 6 months in 223 participants in a community-based NDPP program delivered FTF or by DVD. Stewart et al. [16] recently reported a 6-month weight loss of −3.3 kg in a small sample (n = 33) of primarily men (82%) who completed an NDPP intervention delivered by text message. Interestingly, weight loss across 6 months in the current trial exceeds the ~4% mean weight loss across 12 months reported for meta-analyses of both in-person and technology-based delivery of the NDPP [18,19,20] and the −4.2% weight loss reported from the NDPP national database [4]. In the current trial, the percent of participants achieving the ≥5% weight loss goal (44%) and the percent of participants achieving the ≥150 min/week MVPA goal (75%) in the CE-Zoom^®^ group across 6 months exceeds the results from over 12 months from NDPP national database (36% ≥5% weight loss, 42% ≥150 min/week MVPA [4]). In total, the results from this pilot trial suggest the potential effectiveness of the NDPP Prevent T2 curriculum delivered through CE to reach underserved populations of rural adults to prevent T2D. 

CE has been building successful nutrition education, including the Expanded Food and Nutrition Program (EFNEP), which has helped low-income families and youth improve food security in rural and urban communities since 1969 [8]. CE is also the implementing agency for the Supplemental Nutrition Assistance Program—Education (SNAP-ED), which provides nutrition education to those eligible to receive food assistance benefits. Chronic disease prevention and management programming including Dining with Diabetes, a community-based program with the goal of improving the management of Type 2 diabetes, has successfully been disseminated across four states through CE [21]. Thus, combining the success that CE has with community-based health programming with the academic medical center research community, may provide a novel path to improve the reach of the NDPP. 

The Facebook^®^ group utilized in the current trial, which involved minimal intervention by the lifestyle coach, is thus similar to the online delivery format that is currently eligible to receive approval for delivery of the NDPP through the CDC-NDPP recognition program [22]. Although the weight loss across 6 months in the Facebook^®^ group was small (−1.68 kg, −1.6%, 7% ≥5%) minimal lifestyle coach burden and the low cost of intervention delivery suggest that additional trials to evaluate strategies to improve the effectiveness Facebook^®^ for the delivery of the NDPP to rural residents may be warranted.

Strengths of this trial include high rates of participant retention (~88%), attendance (~76%), and compliance with the self-monitoring protocol (~83%) observed across 6 months in both intervention groups. However, the results from this trial need to be interpreted cautiously within the limitations of a non-randomized, non-powered, pilot trial conducted in a homogenous sample of rural adults in terms of race, ethnicity, and education. Additionally, this trial only evaluated the first 6 months of the 12-month NDPP intervention. An adequately powered, randomized controlled trial assessing the effectiveness of CE delivering the entire 12-month NDPP program should be the focus of future studies.

## 5. Conclusions

In conclusion, this pilot trial demonstrated the potential feasibility and effectiveness of the NDPP delivered by a CE family and consumer science agent in a group remote format (Zoom^®^) to adults with prediabetes living in a rural area. As described previously and shown in Figure 1, part of the national mission of CE is to improve health equity and well-being as part of their role within communities [8]. CE is well known and respected in rural communities. It has the personnel and infrastructure required for delivery of the NDPP to residents of rural areas where the prevalence of T2D is high and access to NDPP or other programs designed to limit the development of T2D is low. Thus, additional adequately powered trials in diverse samples designed to develop and evaluate strategies for using CE for the identification, enrollment, retention, and delivery of the NDPP are warranted and have the potential for the significant public health impact on underserved rural residents. 

## Figures and Tables

**Table 1:** Baseline participant characteristics.

	All Participants	CE-Zoom^®^	Facebook^®^
	*N = 31*	*N = 16*	*N = 15*
*Age* (years) *(m* ± *SD)*	55.1 (12.8)	56.4 (11.1)	53.8 (14.7)
*Sex n, %*			
Male	6 (19%)	4 (25%)	2 (13%)
Female	25 (81%)	12 (75%)	13 (87%)
Minority * n, %	5 (16%)	2 (12.5%)	3 (20%)
*BMI* (kg/m^2^) *(m ± SD)*	36.4 (7.8)	36.9 (5.7)	35.8 (9.9)
*Education (n, %)*			
High school grad or less	2 (7%)	2 (13%)	0 (0%)
Some college	5 (16%)	3 (19%)	2 (13%)
Bachelor’s degree	14 (45%)	4 (25%)	10 (67%)
Graduate degree	6 (19%)	4 (25%)	2 (13%)
*Income (n, %)*			
<$40,000	6 (19%)	4 (25%)	2 (13%)
$40,000–$79,999	9 (29%)	4 (25%)	5 (33%)
>$80,000	15 (48%)	8 (50%)	7 (47%)
*Medical Conditions (n, %)*			
Hypertension	18 (58%)	10 (63%)	8 (53%)
Hyperlipidemia	16 (52%)	10 (63%)	6 (40%)
Coronary artery disease	2 (6%)	1 (6%)	1 (6%)
Mental health	7 (23%)	3 (19%)	4 (27%)

* Non-white and/or Hispanic/Latino, Other/unknown ethnicity.

**Table 2:** Recruitment sources with example quotes.

Group	Quote
***CE-Zoom^®^*** *Extension office (31%)* *Healthcare provider (19%)* *Employer (19%)* *Word of mouth (19%)* *Unknown (12%)*	*“I originally found the ad for it in the newspaper. But then, there was also something at my doctor’s office.*
	*“The extension office sent an email to the [County] human resource director and I got it through her. It was just kind of passed around that way through the grapevine of emails.”* *“Before I signed up for this, when I got the email, I went and talked to [my] doctor. I said, “Have you ever heard about this?” And he pointed to a flyer on his cabinet, and said, “No because I’ve never had anybody go through that.” I’m thinking here I am, somebody that is already on metformin. Why wouldn’t you have promoted that with me? I think you need to work with the doctors in rural areas more.”* *“My doctor actually knew about this program but had never really had a patient that had gone through it. So, he was very excited when he found out at my last physical that I was signed up to do this because he was interested in knowing more about the program. But he did not recommend it.”*
***Facebook^®^*** *Extension office (34%)* *Facebook ad (20%)* *Word of mouth (20%)* *Employer (13%)* *Local ad (13%)*	*“I read about the program in the [local news] paper and I read the e-edition. I’m finding things on the internet and so it was just a matter of time before I found it on [the local] webpage”* *“I found out because [other participant] is my neighbor, and she told me about it and I thought yes, I need to do that. I need to get more exercise, for sure.”* *“I’ve had some friends, mostly on Facebook because I’ve talked about it quite a bit on Facebook”*

**Table 3:** Changes in weight and BMI at 6 months.

	CE-Zoom^®^ (*N* = 14)	Facebook^®^ (*N* = 13)	*p*-Value
Weight (kg)	−6.0 ± 8.0	−1.7 ± 3.3	0.13
% Weight Loss	−5.4 ± 7.4	−1.6 ± 2.4	0.11
BMI (m/kg^2^)	−2.2 ± 2.8	−0.7 ± 1.2	0.14
≥5% weight loss (%)	44	7	0.04 *

* *p* < 0.05.

**Table 4:** Participant response to end study satisfaction survey by intervention group.

*Survey Question*	*Response*	CE-Zoom^®^ *N = 15*	Facebook^®^ *N = 13*
*How do you feel about the overall intervention/program?*	Very satisfied	11 (73)	5 (39)
	Satisfied	1 (7)	6 (46)
	Neutral	1 (7)	2 (15)
	Dissatisfied	2 (13)	0
*Rate interaction with lifestyle coach*	Very satisfied	8 (53)	3 (23)
	Satisfied	4 (27)	6 (46)
	Neutral	3 (20)	3 (23)
	Very dissatisfied	0	1 (8)
*Did your physical activity levels change at all?*	Increase	12 (80)	11 (85)
	Decrease	3 (20)	2 (15)
*Did you make changes to your diet during this program?*	Yes	13 (87)	13 (100)
	No	2 (13)	0
*Did you like the length of the intervention?*	Just right	10 (67)	11 (92)
	Too short	5 (33)	1 (8) *
*Participation in the program caused issues in the following areas:*	Family commitment	2 (13)	1 (7)
	Other	1 (7)	1 (7)
*Did you have internet connectivity issues?*	Yes	3 (20)	1 (7)
	No	12 (80)	12 (93)
*Would you recommend NDPP to a friend or family member?*	Yes	13 (87)	12 (92)
	No	2 (13)	1 (8)

* *N* = 12 total respondents in the Facebook^®^ group for this question.

## Data Availability

The data presented in this study are available on request from the corresponding author.
